# The changes of subtype markers between first and second primary breast cancers

**DOI:** 10.1002/cam4.5979

**Published:** 2023-04-25

**Authors:** Chenyang Li, Zhangyan Lyu, Zhipeng Wang, Chunfang Hao, Yubei Huang, Fengju Song

**Affiliations:** ^1^ Department of Epidemiology and Biostatistics Key Laboratory of Molecular Cancer Epidemiology, Key Laboratory of Breast Cancer Prevention and Therapy, Ministry of Education, National Clinical Research Center for Cancer, Tianjin's Clinical Research Center for Cancer, Tianjin Medical University Cancer Institute and Hospital Tianjin China; ^2^ Department of Breast Oncology Tianjin Medical University Cancer Institute and Hospital Tianjin China

**Keywords:** breast cancer, changes, SPBC, subtype markers, survival

## Abstract

**Background:**

Previous studies investigated the changes of subtype markers [estrogen receptor (ER), progesterone receptor (PR), and human epidermal growth factor receptor 2 (HER2)] in several clinical settings, but not for second primary breast cancer (SPBC) after first primary breast cancer (FPBC).

**Methods:**

A total of 15,390 patients with SPBC were preliminarily selected from the Surveillance, Epidemiology, and End Results Program, and 3777 patients with complete information on three subtype markers in both FPBC and SPBC were included in the final analyses. The changes of subtype markers and their prognostic implications and potential influential factors were well investigated.

**Results:**

The overall change rates of ER, PR, and HER2 between FPBC and SPBC were 23.0% (867/3777), 35.0% (1322/3777), and 18.3% (691/3777), respectively. Gains of ER, PR, and HER2 after negative index markers were 48.7% (364/748), 37.9% (418/1103), and 11.5% (370/3211), while losses of markers after positive index markers were 16.6% (503/3029), 33.8%(904/2674), and 56.7%(321/566). Loss of ER was significantly associated with increased mortality (18.1% vs. 7.9%, *p* < 0.001), while gain of ER was significantly associated with decreased mortality (11.5% vs. 23.2%, *p* < 0.001). Similar results were observed for changes of PR status. However, loss of HER2 was significantly associated with decreased mortality (8.7% vs. 16.3%, *p* = 0.014), and no significant association was observed between the gain of HER2 and the prognosis of SPBC. Multivariate competing risk analyses showed similar results. HER2 status in FPBC, chemotherapy, and radiotherapy was significantly associated with changes of ER/PR (all *p* < 0.05), and no available therapies associated with HER2 change.

**Conclusion:**

The changes of subtype markers are observed in a considerable proportion of patients and has statistically significant prognostic implications. Biopsies should be taken as a routine procedure for better therapy management.

## Graphical abstract

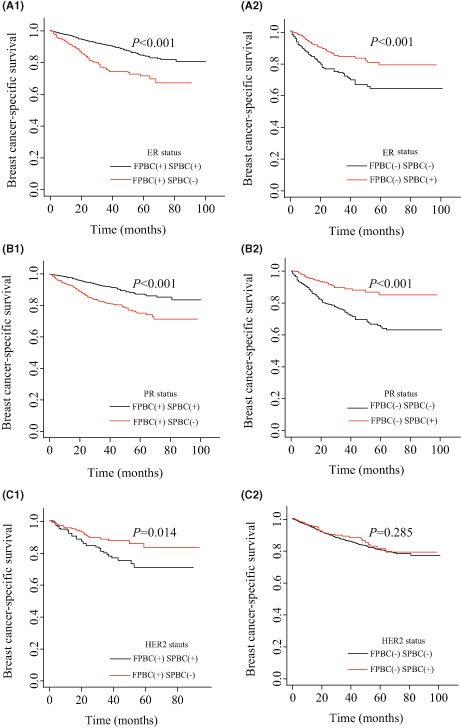

## 1 INTRODUCTION

Female breast cancer has surpassed lung cancer as the leading cause of global cancer incidence in 2020, with an estimated 2.3 million new cases, representing 11.7% of all cancer cases.^1^ Benefiting from population‐wide breast cancer screening and breakthroughs in treatment, mortality of breast cancer gradually decreased over the past decades in several countries.^1^, ^2^, ^3^ However, nearly 12.3% of breast cancer survivors will develop a second primary cancer and suffered a worse prognosis compared to those without a second primary cancer.^4^ Moreover, second primary breast cancers (SPBC) accounted for nearly one‐third of second primary cancers after first primary breast cancer (FPBC).^5^ However, few studies investigate the potential causes of SPBC after the FPBC and explore its role in future cancer management.

Subtype markers of breast cancer, including estrogen receptor (ER), progesterone receptor (PR), and human epidermal growth factor receptor 2 (HER2), have been widely used to guide the clinical treatment of breast cancer.^6^, ^7^ Previous studies have revealed a lack of stability of ER, PR, and HER2 in the clinical setting of relapse, and further suggest significant differential prognosis related to changes of ER and PR.^8^, ^9^, ^10^, ^11^, ^12^ If these therapy‐predictive markers continue to change throughout the second primary cancer, the change would provide important information on the potential therapy‐related lesions (including lesions related to chemotherapy, radiotherapy, and HER2‐targeted therapy) in FPBC, and would also inform potential interaction between different therapies. These clues would enable better therapy management for both FPBC and SPBC.

Therefore, in this study, we aimed to assess the change rates of ER, PR, and HER2 between FPBC and SPBC, investigate its associations with tumor characteristics and prognosis of SPBC, and then ascertain the potential clinical factors which may be related to the changes of these markers.

## 2 METHODS

### 2.1 Study population

The Surveillance, Epidemiology, and End Results (SEER) Program of the National Cancer Institute is a network of population‐based incident tumor registries from geographically distinct regions in the United States.^13^, ^14^ The SEER program collects uniformly reported data on patient demographics, month and year of diagnosis, tumor characteristics, treatment utilization, and mortality for all incident cancers from selected population‐based cancer registries in the United States. The analysis data were downloaded from the SEER database containing information on cancer patients diagnosed from 2000 to 2018, released on April 2021, based on the November 2020 submission [Incidence—SEER Research Plus Data, 18 Registries, Nov 2020 Sub (2000–2018)] by the SEER*Stat version 8.4.0 (SEER ID: 12311‐Nov2021).

Since HER2 receptor status for breast cancer patients was collected from 2010,^15^ breast cancer patients diagnosed in 2010 and later were selected from the SEER program Plus Data and included in this population‐based cohort study. Briefly, a total of 599,399 FPBC patients were initially selected from the SEER program Plus Data. After excluding male patients (*N* = 4106), patients with age at diagnosis younger than 20 or older than 85 (*N* = 23,426), and those with borderline/unknown ER/PR/HER2 (*N* = 136,941), a total of 434,926 female FPBC with complete information on ER, PR, and HER2 were identified. After further excluding patients without secondary primary cancer (*N* = 418,167) and second primary cancer diagnosed within 6 months after FPBC (*N* = 1369), a total of 15,390 second primary cancer patients after FPBC were selected. Finally, after excluding patients with second primary cancer but not breast cancer (*N* = 11,084) and borderline/unknown ER, PR, or HER2 in SPBC (*N* = 529), a total of 3777 SPBC patients with completed information on ER, PR, and HER2 in both FPBC and SPBC were included in the final analyses. Detailed information in data selection is referred in Figure S1.

### 2.2 ER, PR, and HER2 receptor status

SEER registries began to collect data on ER and PR status for breast cancer cases since 1990 and to collect HER2 receptor status since 2010.^15^ The data on ER, PR, and HER2 status were recorded by the SEER program in the following categories: (1) positive (+), (2) negative (−), (3) borderline, (4) unknown, (5) recode not available. To get a clear change in ER, PR, and HER2 status between FPBC and SPBC, only patients with positive or negative biomarkers were included in the final analyses. Hormone receptor (HR) was defined as positive if either ER or PR was positive, and HR was defined as negative if both ER and PR were negative. The derived HER2 summary variable was created by the SEER program using several HER2‐related site‐specific factors from the SEER data collection system. Breast cancer subtypes are defined by joint hormone receptors (ER and PR) and HER2 status.

### 2.3 Follow‐up, SPBC ascertainment, and outcomes

SEER data were linked with data from the National Center for Health Statistics to determine death and cause of death. Follow‐up time, as recorded in months, started at the 6 months after diagnosis of FPBC and ended at the date of death, date last known to be alive, or end of follow‐up (December 31, 2018), whichever came first. The median follow‐up time was 75 months between FPBC and SPBC, and 24 months between diagnosis of SPBC and end of follow‐up. The SEER definition of multiple primary tumors was used to define SPBC in this study, which took into account the site of origin, time since diagnosis, histology, tumor behavior, and laterality of paired organs.^4^ Breast cancer‐specific mortality was considered as the primary outcomes. The changes of each marker between FPBC and SPBC were divided into four patterns: stable positive [FPBC (+)/SPBC (+)], loss of marker [FPBC (+)/SPBC (−)], stable negative [FPBC (−)/SPBC (−)], and gain of marker [FPBC (−)/SPBC (+)].

### 2.4 Statistical Analysis

The overall change rates of subtype markers were calculated as the number of patients with any changes of subtype markers (including either gain or loss of subtype markers) in SPBC divided the total number of patients with FPBC. The gain rates of subtype markers were calculated as the number of patients with gain of positive subtype markers in SPBC divided the number of patients with negative subtype markers in FPBC, while loss rates of subtype markers were calculated as the number of patients with loss of positive subtype markers in SPBC divided the number of patients with positive subtype markers in FPBC. Since the denominator of these three rates is not the same, the overall change rates cannot simply be calculated as the sum of the gain rates and loss rates.

The McNemar's test was used to analyze and compare the changes of ER, PR, and HER2 between FPBC and SPBC. Chi‐square tests were used to compare the proportion of early‐stage SPBC in overall SPBC by change of subtype markers. Early‐stage SPBC was defined as carcinoma in situ and localized breast cancers according to SEER historic stage. Fine‐gray survival curves were used to compare the survival by change of ER, PR, and HER2. After setting deaths from other cancers and non‐cancer deaths as competing risks, multivariate competing risk models were used to investigate the associations between breast cancer‐specific mortality of SPBC and concerning markers' change after adjusting for age at diagnosis(<50, 50–60, ≥60 years), year of diagnosis (≤2015, >2015), registries, race (White, non‐White), marital status (married, other), grade (I + II, III), SEER historic stage (in situ + localized, regional + distant), chemotherapy (no, yes), radiation therapy (no, yes), and surgery (no, yes) in SPBC. HER2 status in SPBC was further adjusted in the multivariate competing risk model for ER/PR change with the prognosis of SPBC, while HR status in SPBC was further adjusted for HER2 change with the prognosis of SPBC. Moreover, multivariate competing risk models were further conducted to investigate whether different treatments were still effective for SPBC patients with different change patterns of markers. Finally, multivariate logistic regression analyses were performed to investigate the potential clinical factors in FPBC associated with the changes of markers. HER2 status in FPBC was further included as a potential factor for the change of ER/PR change in the multivariate logistic regression model, while HR status in FPBC was further included for the HER2 change.

A *p*‐value<0.05 (two‐tailed) was considered as statistically significant. All the statistical analyses were carried out with SAS 9.4 (Statistical Analysis System Institute, Cary, North Carolina, USA) and the figures were drawn by R software (version 4.1.0).

## 3 RESULTS

### 3.1 The changes of subtype markers between FPBC and SPBC

**FIGURE 1:**
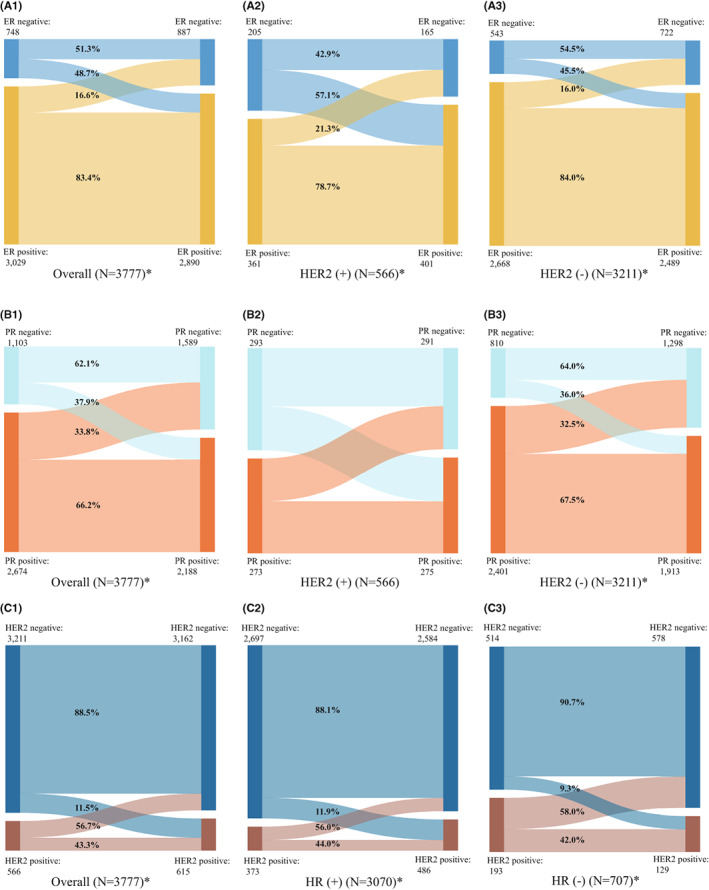
Change of subtype markers between first primary breast cancer (FPBC) and second primary breast cancer (SPBC). ER, estrogen receptor; HER2, human epidermal growth factor receptor‐2; HR, hormone receptor; PR, progesterone receptor. *p value <0.05 for McNemar's tests between FPBC and SPBC. (A1–A3) Changes of ER between FPBC and SPBC; (B1–B3) changes of PR between FPBC and SPBC; (C1–C3) changes of HER2 between FPBC and SPBC.

A total of 3777 patients were finally included in the analysis (Figure S1). As shown in Figure 1 and Table S1, the overall change rates of ER, PR, and HER2 between FPBC and SPBC were 23.0% (867 of 3777), 35.0% (1322 of 3777), and 18.3% (691 of 3777), respectively. Losses of ER, PR, and HER2 in SPBC among patients with positive index markers in FPBC were 16.6% (503/3029), 33.8% (904/2674), and 56.7% (321/566), respectively, while gains of these markers among patients with negative index markers were 48.7% (364/748), 37.9% (418/1103), and 11.5% (370/3211), respectively. Subgroup analyses showed ER or PR change was more pronounced in HER2‐positive FPBC than in HER2‐negative FPBC, while HER2 change was more pronounced in HR‐negative FPBC than in HR‐positive FPBC. McNemar's tests showed that the changes in these markers between intraindividual FPBC and SPBC were significant in the overall population as well as in different subgroups (all *p* values <0.001) (Figure 1 and Table S1).

### 3.2 The changes of subtype markers and early‐stage SPBC

**FIGURE 2:**
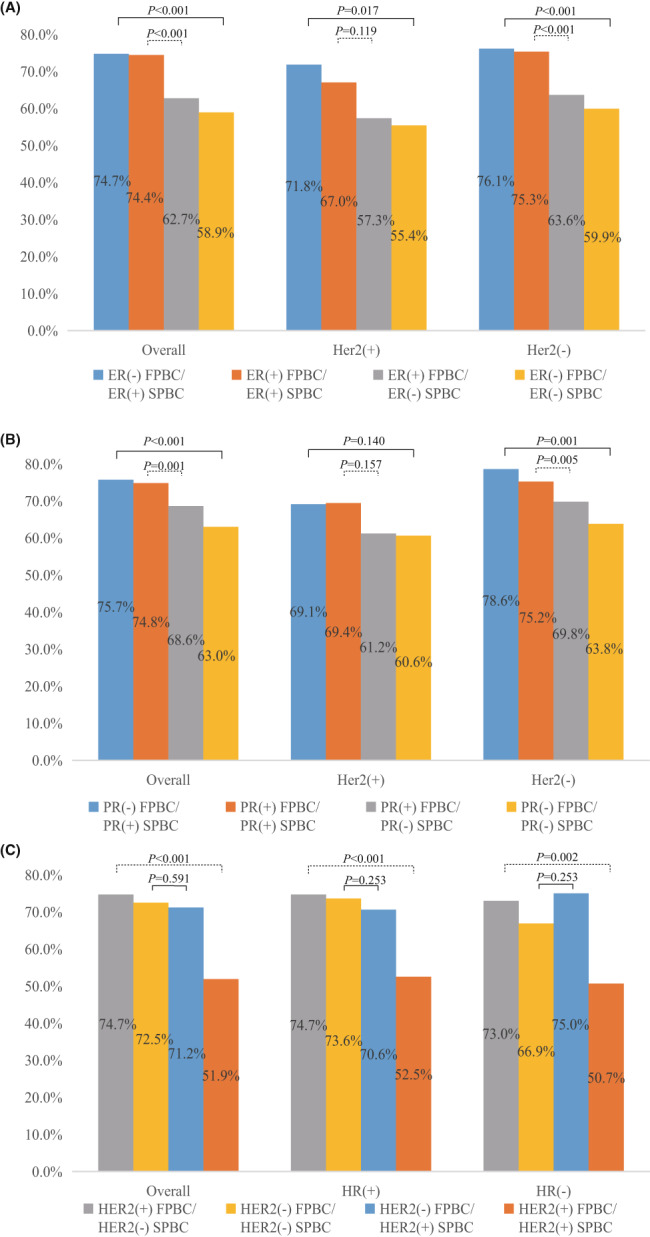
Early‐stage second primary breast cancer (SPBC) by change of subtype markers. ER, estrogen receptor; FPBC, first primary breast cancer; HER2, human epidermal growth factor receptor‐2; HR, hormone receptor; PR, progesterone receptor. (A) changes of ER status; (B) changes of PR status; (C) changes of HER2 status.

As shown in Figure 2 and Table S2, losses of ER and PR were significantly associated with decreased early‐stage SPBC [62.7% (312/498) vs. 74.4% (1842/2477), *p* < 0.001; 68.6% (612/892) vs. 74.8% (1297/1735), *p* < 0.001] compared to stable positive ER/PR, while gains of ER and PR were significantly associated with increased early‐stage SPBC [74.7% (272/364) vs. 58.9% (219/372), *p* < 0.001; 75.7% (315/416) vs. 63.0% (421/668), *p* < 0.001, respectively] compared to stable negative ER/PR. However, loss of HER2 significantly was associated with increased early‐stage SPBC [74.7% (236/316) vs. 51.9% (122/235), *p* < 0.001] compared to stable positive HER2, while gain of HER2 was not significantly associated with early‐stage SPBC [71.2% (262/368) vs. 72.5% (2025/2792), *p* = 0.591] compared to stable negative HER2. Subgroup analyses for both ER/PR change stratified by HER2 status in FPBC and HER2 change stratified by HR status in FPBC showed similar results as observed in the above primary analyses.

### 3.3 The changes of subtype markers and breast cancer‐specific mortality

**FIGURE 3:**
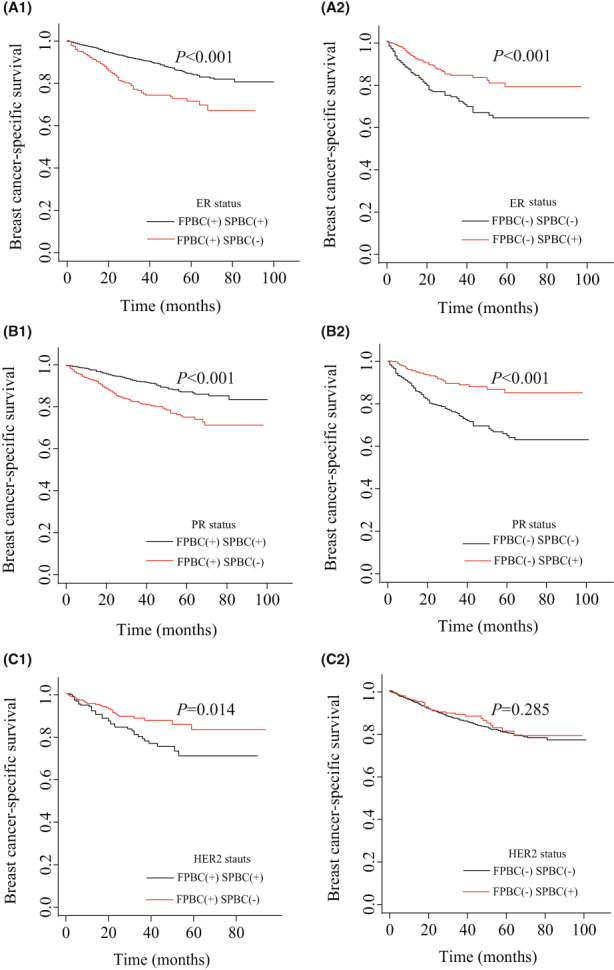
Breast cancer‐specific survival curve of second primary breast cancer (SPBC) by change of subtype markers. ER: estrogen receptor; FPBC: first primary breast cancer; HER2: human epidermal growth factor receptor‐2; PR: progesterone receptor. (A1, A2) ER status in FPBC and SPBC. (B1, B2) PR status in FPBC and SPBC. (C1, C2) HER2 status in FPBC and SPBC.

A total of 411 SPBC patients died of breast cancer at the end of the follow‐up. As shown in Figure 3 and Table S3, compared to stable positive ER/PR, losses of ER and PR were significantly associated with increased breast cancer‐specific mortality in SPBC (ER: 18.1% vs. 7.9%, PR: 14.5% vs. 5.9%, both *p‐*value for Fine‐Grey test <0.001), while gains of ER and PR were significantly associated decreased breast cancer‐specific mortality in SPBC (ER: 11.5% vs. 23.2%, PR: 7.9% vs. 20.8%, both *p‐*value for Fine‐Grey test <0.001). However, loss of HER2 status was significantly associated with decreased breast cancer‐specific mortality in SPBC (8.7% vs. 16.3%, *p* = 0.014), but there was no statistically significant association between gain of HER2 and breast cancer‐specific mortality in SPBC (*p* = 0.285). Subgroup analyses for both ER/PR change stratified by HER2 status in FPBC and HER2 change stratified by HR status in FPBC showed similar results as observed in the above primary analyses (Table S3).

**FIGURE 4:**
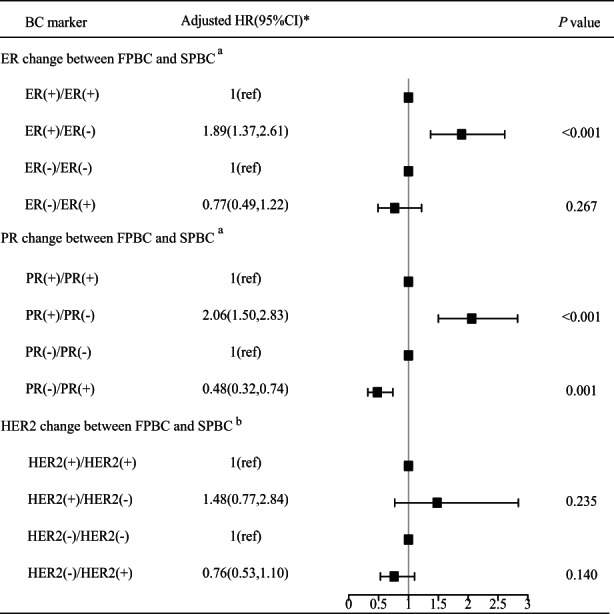
Multivariate competing risk analyses on the breast cancer‐specific mortality of second primary breast cancer (SPBC) by change of subtype markers. ER, estrogen receptor; HER2, human epidermal growth factor receptor‐2; HR, hormone receptor; PR, progesterone receptor. *Age at diagnosis (<50, 50–60, ≥60 years), race (White, non‐White), marital status (married, other), grade (I + II, III), SEER historic stage (in situ + localized, regional + distant), chemotherapy (no, yes), radiation therapy (no, yes), and surgery (no, yes) in the SPBC were adjusted. ^a^HER2 status in the SPBC was further adjusted; ^b^HR status in the SPBC was further adjusted.

After further adjusting the potential clinical factors associated with breast cancer‐specific mortality in the multivariate survival analysis (Figure 4 and Table S4), losses of ER and PR status were significantly associated with an 89% [hazard ratio (HR): 1.89; 95% CI: 1.37–2.61; *p <* 0.001] and 106% (HR: 2.06; 95% CI: 1.50–2.83; *p* < 0.001) increased risk of breast cancer‐specific mortality in SPBC, while gain of PR status was significantly associated with an 46% decreased risk of breast cancer‐specific mortality (HR: 0.48; 95% CI: 0.32–0.74; *p* = 0.001). There was no statistically significant difference in breast cancer‐specific mortality between HER2 change and stable HER2 status after adjusting for other clinical factors.

### 3.4 Therapies management for SPBC with different change patterns of ER and PR

Since the change of HER2 had no impact on the prognosis of SPBC as shown above, we further investigated the effects of different treatments for SPBC by different change patterns of ER/PR. As shown in Table 1, after adjusting for other clinical factors, chemotherapy was significantly associated with decreased risk of breast cancer‐specific mortality for patients with loss of ER (HR: 0.51, 95%CI: 0.29–0.90, *p* = 0.020) or loss of PR (HR: 0.47, 95%CI: 0.28–0.79, *p* = 0.004), respectively, while radiotherapy was not significantly associated with the prognosis of SPBC across all subgroups of patients with different change patterns of ER/PR (all *p* > 0.05). In contrast, surgery can significantly improve the prognosis of SPBC across all subgroups of patients with different change patterns of ER/PR (all *p* < 0.05).

**TABLE 1:** Effects of different treatment for second primary breast cancer (SPBC) by the changes of ER and PR.

BC markers	Chemotherapy		Radiotherapy		Surgery	
	HR (95%CI)^a^	*p* value	HR (95%CI)^a^	*p* value	HR (95%CI)^a^	*p* value
ER change between FPBC and SPBC						
ER(+)/ER(+)	0.78 (0.52,1.17)	0.232	0.89 (0.62,1.27)	0.508	**0.32 (0.20,0.52)**	**<0.001**
ER(+)/ER(−)	**0.51 (0.29,0.90)**	**0.020**	0.68 (0.40,1.16)	0.156	**0.20 (0.10,0.40)**	**<0.001**
ER(−)/ER(−)	0.61 (0.35,1.05)	0.073	1.01 (0.60,1.71)	0.959	**0.34 (0.19,0.60)**	**<0.001**
ER(−)/ER(+)	1.02 (0.45,2.27)	0.971	0.52 (0.24,1.13)	0.098	**0.16 (0.05,0.50)**	**0.002**
PR change between FPBC and SPBC						
PR(+)/PR(+)	0.99 (0.59,1.70)	0.971	0.75 (0.46,1.22)	0.240	**0.37 (0.20,0.70)**	**0.002**
PR(+)/PR(−)	**0.47 (0.28,0.79)**	**0.004**	0.97 (0.64,1.46)	0.871	**0.27 (0.16,0.45)**	**<0.001**
PR(−)/ER(−)	0.58 (0.37,0.90)	0.016	0.93 (0.62,1.40)	0.733	**0.27 (0.17,0.43)**	**<0.001**
PR(−)/PR(+)	1.10 (0.31,3.92)	0.890	0.43 (0.15,1.24)	0.120	**0.21 (0.06,0.71)**	**0.013**

*Note*: Bold indicates statistical differences.

Abbreviation: FPBC, first primary breast cancer.

^a^ Age at diagnosis (<50, 50–60, ≥60 years), race (White, non‐White), HER2 status in the second primary breast cancer, grade (I + II, III), stage (in situ + localized, regional + distant), chemotherapy (no, yes), radiation therapy (no, yes), and surgery (no, yes) were initially included in the multivariate competing risk analyses.

### 3.5 Potential clinical factors in FPBC associated with the changes of subtype markers

As shown in Tables S5–S7, the change rates of subtype markers were significantly different across several subgroups stratified by age at diagnosis or race. After adjusting these potential non‐interventional factors in FPBC, as shown in Table 2, positive HER2 status in FPBC was significantly associated with increased risk of gain of ER (OR:2.05, 95%CI: 1.36–3.08; *p =* 0.001), the gain of PR (OR:1.83, 95%CI: 1.31–2.55; *p <* 0.001), and loss of PR (OR: 1.46, 95%CI: 1.06–2.02; *p* = 0.020). Chemotherapy in FPBC was significantly associated with an increased risk of loss of ER (OR: 1.76, 95%CI: 1.31–2.37; *p* < 0.001), loss of PR (OR: 1.61, 95%CI: 1.23–2.12; *p* = 0.001), and decreased risk of gain of ER (OR: 0.69, 95%CI: 0.49–0.96, *p* = 0.026). Radiotherapy was significantly associated with an increased risk of loss of ER (OR: 1.32, 95%CI: 1.04–1.68; *p* = 0.022) and loss of PR (OR: 1.24, 95%CI: 1.02–1.52; *p* = 0.033). However, no available therapies or interventional factors in FPBC, including positive HR, chemotherapy, radiotherapy, and surgery, were significantly associated with the changes of HER2 status (all *p* > 0.05).

**TABLE 2:** Multivariate logistic analyses on the associations between change of subtype markers and selected clinical factors in first primary breast cancer (FPBC).

Selected clinical factors in FPBC	OR (95%CI)^a^			
	FPBC (+) SPBC(−) versus FPBC (+) SPBC(+)	*p* value	FPBC (−) SPBC(+) versus FPBC (−) SPBC(−)	*p* value
Change of ER status^b^				
HER2 status (Pos vs. Neg)	1.11 (0.80,1.56)	0.536	**2.05 (1.36,3.08)**	**0.001**
Chemotherapy (Yes vs. No)	**1.76 (1.31,2.37)**	**<0.001**	0.92 (0.60,1.40)	0.694
Radiotherapy (Yes vs. No)	**1.32 (1.04,1.68)**	**0.022**	1.05 (0.72,1.53)	0.818
Surgery (Yes vs. No)	1.32 (0.58,3.03)	0.51	0.99 (0.35,2.84)	0.994
Change of PR status^b^				
HER2 status (Pos vs. Neg)	**1.46 (1.06,2.02)**	**0.020**	**1.83 (1.31,2.55)**	**<0.001**
Chemotherapy (Yes vs. No)	**1.61 (1.23,2.12)**	**0.001**	**0.69 (0.49,0.96)**	**0.026**
Radiotherapy (Yes vs. No)	**1.24 (1.02,1.52)**	**0.033**	1.05 (0.77,1.44)	0.757
Surgery (Yes vs. No)	1.10 (0.57,2.10)	0.782	0.97 (0.37,2.55)	0.952
Change of HER2 status^c^				
HR status (Pos vs. Neg)	0.93 (0.58,1.47)	0.742	1.39 (0.92,2.12)	0.121
Chemotherapy (Yes vs. No)	1.13 (0.69,1.88)	0.626	1.10 (0.77,1.56)	0.603
Radiotherapy (Yes vs. No)	0.81 (0.52,1.26)	0.346	1.12 (0.87,1.46)	0.381
Surgery (Yes vs. No)	1.78 (0.63,5.05)	0.278	1.75 (0.53,5.72)	0.358

*Note*: Bold indicates statistical differences.

Abbreviations: ER, estrogen receptor; HER2, human epidermal growth factor receptor‐2; HR, hormone receptor; OR (95% CI): odds ratio (95% confidence interval); PR, progesterone receptor; SPBC, second primary breast cancer.

^a^ Age at diagnosis (<50, 50–60, ≥60 years), race (White, non‐White), grade (I + II, III), SEER historic stage (in situ + localized, regional + distant), chemotherapy (no, yes), radiation therapy (no, yes), and surgery (no, yes) in the FPBC were adjusted.

^b^ HER2 status in the FPBC was further adjusted.

^c^ HR status in the FPBC was further adjusted.

## 4 DISCUSSION

To our knowledge, this is the first study to investigate the changes of subtype markers (including ER, PR, and HER2 status) between FPBC and SPBC. In this study, we found a considerable proportion of patients had experienced changes of ER, PR, and HER2 status in SPBC after FPBC, respectively. The changes of subtype markers not only significantly relate to the tumor characteristics of SPBC, but also has statistically significant prognostic implications. Moreover, therapies in FPBC, such as chemotherapy and radiotherapy, may induce changes in subtype markers.

Previous studies had revealed the changes of ER, PR, and HER2 in several clinical settings, including neoadjuvant chemotherapy,^16^, ^17^, ^18^ relapse, multiple consecutive relapses, metastasis, and multiple consecutive metastases.^19^, ^20^, ^21^ The overall change rates varied from 2.5% to 17% for ER status, 5.9% to 51.7% for PR status, and 2% to 13% for HER2 status.^18^ The changes rates of these markers observed in this study were similar with previous results in the settings of relapse, multiple consecutive relapses, metastasis, and multiple consecutive metastases, and were relatively higher than those in the settings of neoadjuvant chemotherapy. These results would suggest that the effect of neoadjuvant chemotherapy on these therapy‐predictive markers is likely to be a long‐term cumulative process. Detecting considerable changes in these markers immediately after neoadjuvant chemotherapy is relatively unlikely since stable tumor clones have not yet formed. Therefore, changes in these markers can only be detected in a minority of patients in the setting of neoadjuvant therapy, while the changes can be detected in a relatively large proportion of patients in the settings of relapse, metastasis, and second primary cancer. Additionally, due to survivor bias, we cannot observe the changes in breast cancer patients who have died, so the change observed in breast cancer survivors likely underestimate the overall change in all breast cancer patients in real‐world settings. Compared to sporadic breast cancer, genetic mutations in familial hereditary cancers play a stronger role in cancer development, while more similar genetic basis and phenotype (such as subtype markers) between FPBC and SPBC are presumed to be observed in hereditary cancers than sporadic cancers. Conversely, for sporadic breast cancer, more different genetic basis and phenotypes are presumed to exist between FPBC and SPBC. However, if FPBC and SPBC occurred in the same individual, it is undeniable that there would be a certain relationship between the two cancers. Although the association between sporadic mutations and SPBC would not be as strong as the association between familial inherited mutations and SBPC, mutations of FPBC and SPBC are generally presumed to be related with each other even if they come from relatively independent cancers. As the genetic mutations between FPBC and SPBC within the same individual, their phenotypes (such as subtype markers) are also presumed to be related with each other even if they come from sporadic FPBC and SPBC. Therefore, either the consistent and inconsistent genetic mutation or the genetic and phenotypic changes between sporadic FPBC and SPBC deserve further studies.

The prognostic implications of this changes are very important. As observed in previous studies,^11^ patients with a gain of HR will have a better prognosis than those with stable HR, while loss of HR will introduce to a worsening prognosis on the survival. Several explanations can be found for these prognostic implications. Loss of HR generally means no response to endocrine therapy; thus, these patients would not benefit from endocrine in therapy. Conversely, gain of HR would introduce additional choices of endocrine therapy, potentially leading to tumor response and prolonged survival in some patients.^11^ However, similar to previous studies,^8^, ^10^ change of HER2 was not found to be significantly associated with BC prognosis after adjusting other clinical factors. Several reasons could lead to this result. The most important reason should be the relatively low change rate of HER2 (18.3%, with 23.0% and 35.0% for ER and PR, respectively) resulting relatively small sample size of patients with HER2 change. After multivariable adjustment, this weak change is not enough to independently affect the patient's prognosis. Second, among patients with change of HER2, nearly half of the change was loss of HER2. Loss of HER2 means loss of the chance of targeted therapy. Moreover, the remaining change of HER2, namely gain of HER2, was found to be not significantly associated with early‐stage SPBC, which suggested that patients with gain of HER2 would difficultly benefit from the current therapies. Third, among breast cancer patients without HER2 changes, most of them have persistently negative HER2, and they will not be provided with regular HER2‐targeted therapy. The remaining patients with persistently positive for HER2 would probably not benefit from the second‐round HER2 treatment after first‐round therapy for the FPBC due to potential drug resistance. All of these would collectively lead to the non‐significant association between HER2 change and prognosis. Additionally, all these changes of subtype markers would also demonstrate intratumor heterogeneity with various progression capacities in primary breast tumors. Inherent host and tumor biologic factors may also be involved in the clonal expansion and therapy response capacities before and after HER2 change.^11^

Importantly, until now, the factors associated with the changes of ER, PR, and HER2 are still unclear. As mentioned above, neoadjuvant chemotherapy may affect the change of marker status.^16^, ^17^, ^18^ Besides, radiotherapy might promote the loss of ER/PR.^22^ Similarly, in our study, both chemotherapy and radiotherapy in FPBC were significantly associated with the changes in ER and PR status. More importantly, compared to radiotherapy, chemotherapy appears more likely to induce changes in HR, either gain of HR or loss of HR. Although it's difficult to address why chemotherapy could induce gain of HR, one potential explanation deserve attention. For patients with borderline or low ER/PR expression, which were generally defined as negative HR, if their first cancer benefits from chemotherapy, they might have a higher chance of having the same chemotherapy in their second cancer. In this case, if these patients obtain positive HR expression, chemotherapy is basically considered as a potential causal of the gain of HR. Although we cannot address which ingredients in chemotherapy are more likely to induce the changes in HR due to limited data, better chemotherapy management should deserve more attention than radiotherapy management. Positive HER2 status in FPBC was also significantly associated with the changes in HR, which may suggest the potential interactions between different therapies. Unfortunately, we did not find any available therapies or interventional factors that might affect the change in HER2 status. It is unclear whether HER2‐targeted therapy is the only clinical interventional factor associated with HER2 alterations. Moreover, we found that the change rates of subtype markers were significantly different across several subgroups stratified by age at diagnosis or race (Table S3–S5), which suggested that non‐interventional factors should also be considered in the better therapy management. Additionally, treatment‐induced clonal selection, true biological modifications, preanalytical, analytical pitfalls, sampling errors, and tumor heterogeneity would also represent some of the possible explanations.^21^

In addition to the strengths and clinical implications mentioned above, limitations in this study also deserve attention. First, ER and PR status in SEER were assessed by immunohistochemistry (IHC), while HER2 was derived with information from IHC, fluorescence in situ hybridization (FISH) test, and chromogenic in situ hybridization (CISH) test. These markers status has not been confirmed by other methods, such as immunocytochemistry (ICC), which would lead to discordance between different methods. However, previous studies suggested that results based on biochemical receptor determinations, IHC/ICC, and other methods revealed a relatively high concordance value.^11^, ^23^ Second, as mentioned above, we cannot address which ingredients in chemotherapy are more likely to induce the changes in HR due to limited data. However, this limitation cannot change the overall conclusion of this study, and it also does not affect our overall recommendation that better chemotherapy management should deserve more attention than radiotherapy management. Furthermore, due to lack of information of neoadjuvant and/or adjuvant endocrine therapy and HER2‐targeted therapy in SEER database, further studies are urgently needed to investigate whether the changes of HR/HER2 status are associated with the therapies, respectively. Third, since the study population was only selected from the US SEER cohort, there was no validation in other populations. Therefore, there may be some limitations in extrapolating the current results to other population. However, this is the largest sample ever studied. Finally, missing values of index variables may not be at random at different SEER registries and different period and would bias the current results. To solve this problem, we first adjusted the SEER registries and year of diagnosis in the multivariable Cox competing risk regression. Additionally, we had presented and compared the clinical characteristics of overall FPBC patients, FPBC patients without second primary cancer (SPC), SPC patients exclude SPBC, and SPBC (Table S8). Although there were some differences among different populations, the absolute percentage difference between any subgroup within any index variable did not exceed 3% (Table S8). These results suggested that there was no obvious selection bias in clinical characteristics between selected and unselected populations.

In conclusion, the changes of subtype markers are observed in a considerable proportion of patients and the change rates are likely to be underestimated. Since the changes of subtype markers are not only observed after neoadjuvant chemotherapy, but also in the settings of relapse, metastasis, and second primary cancer, these results suggest that the changes of subtype markers would probably change throughout tumor progression and have statistically significant prognostic implications. Therefore, biopsies should be taken as a routine procedure for better therapy management. Moreover, due to the potential limitations, further studies with more sophisticated design and larger sample size are needed to validate these results.

## AUTHOR CONTRIBUTIONS

**Chenyang Li:** Formal analysis (equal); investigation (equal); writing – original draft (equal); writing – review and editing (equal). **Zhangyan Lyu:** Writing – review and editing (equal). **Zhipeng Wang:** Writing – review and editing (equal). **Chunfang Hao:** Writing – review and editing (equal). **Yubei Huang:** Funding acquisition (equal); supervision (equal); writing – review and editing (equal). **Fengju Song:** Funding acquisition (equal); supervision (equal); writing – review and editing (equal). All authors approved the final version.

## FUNDING INFORMATION

This work was supported by the Chinese National Key Research and Development Project (No. 2021YFC2500400); Tianjin Municipal Health Committee Foundation (No.TJWJ2021MS008), Tianjin Science and Technology Committee Foundation (No.18JCQNJC80300).

## CONFLICT OF INTEREST STATEMENT

The authors declare that they have no competing interests.

## Supporting information


Data S1.

## Data Availability

The datasets used and/or analyzed during the current study are available from the Surveillance, Epidemiology, and End Results (SEER) Program of the National Cancer Institute.
