# Supplementary material for: The changes of subtype markers between first and second primary breast cancers
Source: Cancer Med. 2023 Apr 25;12(12):13649–60. doi: 10.1002/cam4.5979 (PMC10315743; doi:10.1002/cam4.5979)
Supplement: Supplementary file 1 — Data S1. [file CAM4-12-13649-s001.docx]

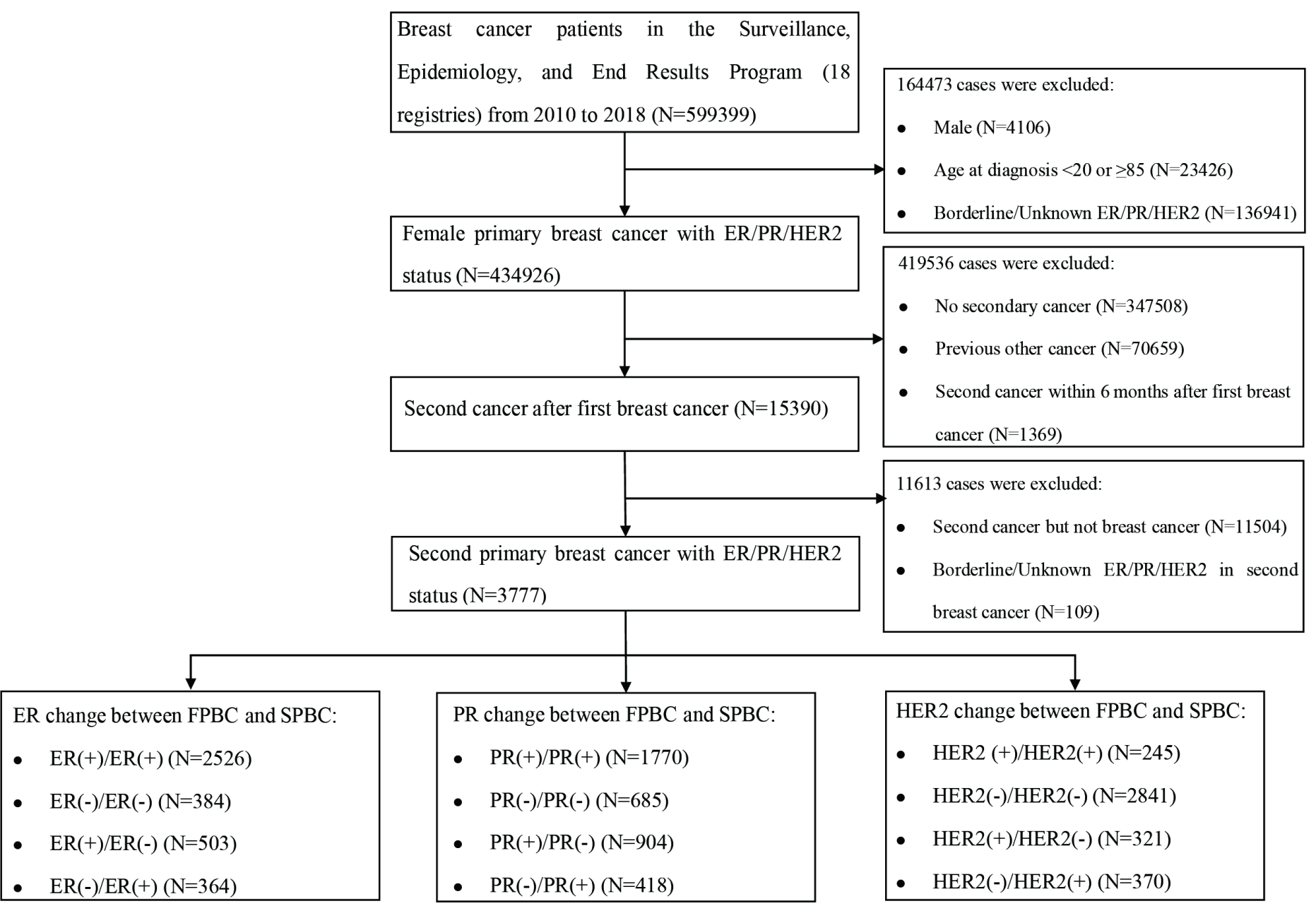


**Figure S1. Flowchart of patient selection in the study.** Note: ER, estrogen receptor; PR, progesterone receptor; HER2, human epidermal growth factor receptor-2.

**Table S1. Change of subtype markers between first primary breast cancer (FPBC) and second primary breast cancer (SPBC).**

| FPBC | | | | | |  | SPBC | | | *P-*value^*^ |
| --- | --- | --- | --- | --- | --- | --- | --- | --- | --- | --- |
| Markers | N | % | Markers | N | % |  | Markers | N | % |  |
| ER status |  |  |  |  |  |  |  |  |  |  |
| Overall | 3777 | 100.0% | ER(+) | 3029 | 80.2% |  | ER(+) | 2526 | 83.4% | **<0.001** |
|  |  |  |  |  |  |  | ER(-) | 503 | 16.6% |  |
|  |  |  | ER(-) | 748 | 19.8% |  | ER(+) | 364 | 48.7% |  |
|  |  |  |  |  |  |  | ER(-) | 384 | 51.3% |  |
| Her2(+) | 566 | 15.0% | ER(+) | 361 | 63.8% |  | ER(+) | 284 | 78.7% | **0.004** |
|  |  |  |  |  |  |  | ER(-) | 77 | 21.3% |  |
|  |  |  | ER(-) | 205 | 36.2% |  | ER(+) | 117 | 57.1% |  |
|  |  |  |  |  |  |  | ER(-) | 88 | 42.9% |  |
| Her2(-) | 3211 | 85.0% | ER(+) | 2668 | 83.1% |  | ER(+) | 2242 | 84.0% | **<0.001** |
|  |  |  |  |  |  |  | ER(-) | 426 | 16.0% |  |
|  |  |  | ER(-) | 543 | 16.9% |  | ER(+) | 247 | 45.5% |  |
|  |  |  |  |  |  |  | ER(-) | 296 | 54.5% |  |
| PR status |  |  |  |  |  |  |  |  |  |  |
| Overall | 3777 | 100% | PR(+) | 2674 | 70.8% |  | PR(+) | 1770 | 66.2% | **<0.001** |
|  |  |  |  |  |  |  | PR(-) | 904 | 33.8% |  |
|  |  |  | PR(-) | 1103 | 29.2% |  | PR(+) | 418 | 37.9% |  |
|  |  |  |  |  |  |  | PR(-) | 685 | 62.1% |  |
| Her2(+) | 566 | 15.0% | PR(+) | 273 | 48.2% |  | PR(+) | 149 | 54.6% | 0.899 |
|  |  |  |  |  |  |  | PR(-) | 124 | 45.4% |  |
|  |  |  | PR(-) | 293 | 51.8% |  | PR(+) | 126 | 43.0% |  |
|  |  |  |  |  |  |  | PR(-) | 167 | 57.0% |  |
| Her2(-) | 3211 | 85.0% | PR(+) | 2401 | 74.8% |  | PR(+) | 1621 | 67.5% | **<0.001** |
|  |  |  |  |  |  |  | PR(-) | 780 | 32.5% |  |
|  |  |  | PR(-) | 810 | 25.2% |  | PR(+) | 292 | 36.0% |  |
|  |  |  |  |  |  |  | PR(-) | 518 | 64.0% |  |
| HER2 status |  |  |  |  |  |  |  |  |  |  |
| Overall | 3777 | 100.0% | HER2(+) | 566 | 15.0% |  | HER2(+) | 245 | 43.3% | **<0.001** |
|  |  |  |  |  |  |  | HER2(-) | 321 | 56.7% |  |
|  |  |  | HER2(-) | 3211 | 85.0% |  | HER2(+) | 370 | 11.5% |  |
|  |  |  |  |  |  |  | HER2(-) | 2841 | 88.5% |  |
| HR(+) | 3070 | 81.3% | HER2(+) | 373 | 12.1% |  | HER2(+) | 164 | 44.0% | **<0.001** |
|  |  |  |  |  |  |  | HER2(-) | 209 | 56.0% |  |
|  |  |  | HER2(-) | 2697 | 87.9% |  | HER2(+) | 322 | 11.9% |  |
|  |  |  |  |  |  |  | HER2(-) | 2375 | 88.1% |  |
| HR(-) | 707 | 18.7% | HER2(+) | 193 | 27.3% |  | HER2(+) | 81 | 42.0% | **<0.001** |
|  |  |  |  |  |  |  | HER2(-) | 112 | 58.0% |  |
|  |  |  | HER2(-) | 514 | 72.7% |  | HER2(+) | 48 | 9.3% |  |
|  |  |  |  |  |  |  | HER2(-) | 466 | 90.7% |  |

Note: *, *P* value for McNemar's tests. ER, estrogen receptor; PR, progesterone receptor; HER2, human epidermal growth factor receptor-2; HR, hormone receptor.

**Table S2. Early-stage second primary breast cancer (SPBC) by change of subtype markers.**

| BC markers in FPBC | | BC markers in SPBC | | Early-stage SPBC | | *P* value |
| --- | --- | --- | --- | --- | --- | --- |
|  |  |  |  | N | % |  |
| ER status |  |  |  |  |  |  |
| Overall | ER(+) | ER(+) | 2477 | 1842 | 74.4% | **<0.001** |
|  |  | ER(-) | 498 | 312 | 62.7% |  |
|  | ER(-) | ER(+) | 364 | 272 | 74.7% | **<0.001** |
|  |  | ER(-) | 372 | 219 | 58.9% |  |
| Her2(+) | ER(+) | ER(+) | 276 | 185 | 67.0% | 0.119 |
|  |  | ER(-) | 75 | 43 | 57.3% |  |
|  | ER(-) | ER(+) | 117 | 84 | 71.8% | **0.017** |
|  |  | ER(-) | 83 | 46 | 55.4% |  |
| Her2(-) | ER(+) | ER(+) | 2201 | 1657 | 75.3% | **<0.001** |
|  |  | ER(-) | 423 | 269 | 63.6% |  |
|  | ER(-) | ER(+) | 247 | 188 | 76.1% | **<0.001** |
|  |  | ER(-) | 289 | 173 | 59.9% |  |
| PR status |  |  |  |  |  |  |
| Overall | PR(+) | PR(+) | 1735 | 1297 | 74.8% | **0.001** |
|  |  | PR(-) | 892 | 612 | 68.6% |  |
|  | PR(-) | PR(+) | 416 | 315 | 75.7% | **<0.001** |
|  |  | PR(-) | 668 | 421 | 63.0% |  |
| Her2(+) | PR(+) | PR(+) | 144 | 100 | 69.4% | 0.157 |
|  |  | PR(-) | 121 | 74 | 61.2% |  |
|  | PR(-) | PR(+) | 126 | 87 | 69.1% | 0.140 |
|  |  | PR(-) | 160 | 97 | 60.6% |  |
| Her2(-) | PR(+) | PR(+) | 1591 | 1197 | 75.2% | **0.005** |
|  |  | PR(-) | 771 | 538 | 69.8% |  |
|  | PR(-) | PR(+) | 290 | 228 | 78.6% | **0.001** |
|  |  | PR(-) | 508 | 324 | 63.8% |  |
| HER2 status |  |  |  |  |  |  |
| Overall | HER2+ | HER2(+) | 235 | 122 | 51.9% | **<0.001** |
|  |  | HER2(-) | 316 | 236 | 74.7% |  |
|  | HER2- | HER2(+) | 368 | 262 | 71.2% | 0.591 |
|  |  | HER2(-) | 2792 | 2025 | 72.5% |  |
| HR (+) | HER2+ | HER2(+) | 158 | 83 | 52.5% | **<0.001** |
|  |  | HER2(-) | 205 | 155 | 74.7% |  |
|  | HER2- | HER2(+) | 320 | 226 | 70.6% | 0.253 |
|  |  | HER2(-) | 2333 | 1718 | 73.6% |  |
| HR (-) | HER2+ | HER2(+) | 77 | 39 | 50.7% | **0.002** |
|  |  | HER2(-) | 111 | 81 | 73.0% |  |
|  | HER2- | HER2(+) | 48 | 36 | 75.0% | 0.253 |
|  |  | HER2(-) | 459 | 307 | 66.9% |  |

Note: *, *P* value for Chi-square tests. FPBC, first primary breast cancer; ER, estrogen receptor; PR, progesterone receptor; HER2, human epidermal growth factor receptor-2; HR, hormone receptor.

**Table S3. Mortality of second primary breast cancer (SPBC) by change of subtype markers.**

| BC markers in FPBC | | BC markers in SPBC | | BC Mortality | | *P* value* |
| --- | --- | --- | --- | --- | --- | --- |
|  |  |  |  | N | % |  |
| ER status |  |  |  |  |  |  |
| Overall | ER(+) | ER(+) | 2526 | 189 | 7.9% | **<0.001** |
|  |  | ER(-) | 503 | 91 | 18.1% |  |
|  | ER(-) | ER(+) | 364 | 42 | 11.5% | **<0.001** |
|  |  | ER(-) | 384 | 89 | 23.2% |  |
| Her2(+) | ER(+) | ER(+) | 284 | 21 | 7.4% | **<0.001** |
|  |  | ER(-) | 77 | 17 | 22.1% |  |
|  | ER(-) | ER(+) | 117 | 11 | 9.4% | **0.010** |
|  |  | ER(-) | 88 | 19 | 21.6% |  |
| Her2(-) | ER(+) | ER(+) | 2242 | 168 | 7.5% | **<0.001** |
|  |  | ER(-) | 426 | 74 | 17.4% |  |
|  | ER(-) | ER(+) | 247 | 31 | 12.6% | **0.001** |
|  |  | ER(-) | 296 | 70 | 23.7% |  |
| PR status |  |  |  |  |  |  |
| Overall | PR(+) | PR(+) | 1770 | 104 | 5.9% | **<0.001** |
|  |  | PR(-) | 904 | 131 | 14.5% |  |
|  | PR(-) | PR(+) | 418 | 33 | 7.9% | **<0.001** |
|  |  | PR(-) | 685 | 143 | 20.8% |  |
| Her2(+) | PR(+) | PR(+) | 149 | 6 | 4.0% | **<0.001** |
|  |  | PR(-) | 124 | 19 | 15.3% |  |
|  | PR(-) | PR(+) | 126 | 8 | 6.4% | **<0.001** |
|  |  | PR(-) | 167 | 35 | 21.0% |  |
| Her2(-) | PR(+) | PR(+) | 1621 | 98 | 6.1% | **<0.001** |
|  |  | PR(-) | 780 | 112 | 14.4% |  |
|  | PR(-) | PR(+) | 292 | 25 | 8.6% | **<0.001** |
|  |  | PR(-) | 518 | 108 | 20.9% |  |
| HER2 status |  |  |  |  |  |  |
| Overall | HER2+ | HER2(+) | 245 | 40 | 16.3% | **0.014** |
|  |  | HER2(-) | 321 | 28 | 8.7% |  |
|  | HER2- | HER2(+) | 370 | 35 | 9.5% | 0.285 |
|  |  | HER2(-) | 2841 | 308 | 10.8% |  |
| HR (+) | HER2+ | HER2(+) | 164 | 28 | 17.1% | **0.002** |
|  |  | HER2(-) | 209 | 12 | 5.7% |  |
|  | HER2- | HER2(+) | 322 | 32 | 9.9% | 0.886 |
|  |  | HER2(-) | 2375 | 220 | 9.3% |  |
| HR (-) | HER2+ | HER2(+) | 81 | 12 | 14.8% | 0.946 |
|  |  | HER2(-) | 112 | 16 | 14.3% |  |
|  | HER2- | HER2(+) | 48 | 3 | 6.3% | **0.032** |
|  |  | HER2(-) | 466 | 88 | 18.9% |  |

Note: *, *P* value for Fine-Gray tests. FPBC, first primary breast cancer; ER, estrogen receptor; PR, progesterone receptor; HER2, human epidermal growth factor receptor-2; HR, hormone receptor.

**Table S4. Multivariate** **competitive risk analyses on the mortality of second primary breast cancer (SPBC).**

| BC marker | Unadjusted  HR (95%CI) | *P* value | Age-adjusted  HR (95%CI) | *P* value ^c^ | Adjusted  HR (95%CI)* | *P* value ^c^ |
| --- | --- | --- | --- | --- | --- | --- |
| ER status ^a^ |  |  |  |  |  |  |
| FPBC(+) |  |  |  |  |  |  |
| SPBC(+) | 1(ref) |  | 1(ref) |  | 1(ref) |  |
| SPBC(-) | 2.50(1.95, 3.22) | <0.001 | **2.46(1.91,3.17)** | **<0.001** | **1.89(1.38,2.60)** | **<0.001** |
| FPBC(-) |  |  |  |  |  |  |
| SPBC(+) | 0.47(0.33, 0.68) | <0.001 | **0.49(0.34,0.71)** | **<0.001** | 0.88(0.57,1.38) | 0.585 |
| SPBC(-) | 1(ref) |  | 1(ref) |  | 1(ref) |  |
| PR status ^a^ |  |  |  |  |  |  |
| FPBC(+) |  |  |  |  |  |  |
| SPBC(+) | 1(ref) |  | 1(ref) |  | 1(ref) |  |
| SPBC(-) | 2.49(1.93, 3.22) | <0.001 | **2.62(2.02,3.41)** | **<0.001** | **1.97(1.45,2.68)** | **<0.001** |
| FPBC(-) |  |  |  |  |  |  |
| SPBC(+) | 0.36(0.24, 0.52) | <0.001 | **0.37(0.26,0.55)** | **<0.001** | **0.54(0.36,0.81)** | **0.003** |
| SPBC(-) | 1(ref) |  | 1(ref) |  | 1(ref) |  |
| HER2 status ^b^ |  |  |  |  |  |  |
| FPBC(+) |  |  |  |  |  |  |
| SPBC(+) | 1(ref) |  | 1(ref) |  | 1(ref) |  |
| SPBC(-) | 0.55(0.34, 0.89) | 0.014 | **0.56(0.34,0.93)** | **0.024** | 1.36(0.75,2.46) | 0.317 |
| FPBC(-) |  |  |  |  |  |  |
| SPBC(+) | 0.83(0.59, 1.17) | 0.285 | 0.80(0.57,1.14) | 0.220 | 0.78(0.54,1.13) | 0.182 |
| SPBC(-) | 1(ref) |  | 1(ref) |  | 1(ref) |  |

Note: FPBC, first primary breast cancer. *, age at diagnosis (<50, 50-60, ≥60 years), year of diagnosis (≤2015, >2015), registries, race (white, non-white), marital status (married, other), grade (I + II, III), SEER historic stage (in situ + localized, regional + distant), chemotherapy (no, yes), radiation therapy (no, yes) and surgery (no, yes) in the SPBC were initially included in the multivariate analyses. a, HER2 status in the SPBC was further adjusted; b, HR status in the SPBC was further adjusted. c, *P* values for competing mortality risks of breast cancer after adjusting other risks of mortality. ER, estrogen receptor; PR, progesterone receptor; HER2, human epidermal growth factor receptor-2; HR, hormone receptor.

**Table S5. Multivariate** **logistic analyses on the factors associated with change of ER status.**

| Characteristics of  FPBC* | FPBC (+), N (%) | | OR (95%CI) | *P* value | FPBC (-), N (%) | | OR (95%CI) | *P* value |
| --- | --- | --- | --- | --- | --- | --- | --- | --- |
|  | SPBC(+) (Ref.) | SPBC(-) |  |  | SPBC(+) | SPBC(-) (Ref.) |  |  |
| Age at diagnosis |  |  |  |  |  |  |  |  |
| <50 years | 535(21.2) | 113(22.5) | 1(ref) |  | 138(35.9) | 58(15.9) | 1(ref) |  |
| 50-60 years | 577(22.8) | 144(28.6) | 1.23(0.90,1.68) | 0.201 | 110(28.7) | 100(27.5) | **2.44(1.50,3.99)** | **<0.001** |
| ≥ 60 years | 1414(56.0) | 246(48.9) | 1.05(0.78,1.41) | 0.747 | 136(35.4) | 206(56.6) | **3.96(2.50,6.25)** | **<0.001** |
| Race |  |  |  |  |  |  |  |  |
| White | 1981(78.5) | 354(70.4) | 1(ref) |  | 225(58.6) | 267(74.0) | 1(ref) |  |
| Non-white | 543(21.5) | 149(29.6) | **1.56(1.22,2.00)** | **<0.001** | 159(41.4) | 94(26.0) | **0.52(0.35,0.76)** | **0.001** |
| Grade |  |  |  |  |  |  |  |  |
| I + II | 1895(79.4) | 327(68.3) | 1(ref) |  | 79(22.1) | 85(24.6) | 1(ref) |  |
| III | 490(20.6) | 152(31.7) | **1.38(1.06,1.79)** | **0.017** | 278(77.9) | 261(75.4) | 0.87(0.57,1.31) | 0.500 |
| SEER historic stage |  |  |  |  |  |  |  |  |
| In situ + Localized | 1888(75.0) | 343(68.5) | 1(ref) |  | 223(58.2) | 223(61.4) | 1(ref) |  |
| Regional + Distant | 630(25.0) | 158(31.5) | 0.73(0.52,1.02) | 0.068 | 160(41.8) | 140(38.6) | 0.99(0.64,1.55) | 0.98 |
| HER2 status |  |  |  |  |  |  |  |  |
| Negative | 2242(88.8) | 426(84.7) | 1(ref) |  | 296(77.1) | 247(67.9) | 1(ref) |  |
| Positive | 284(11.2) | 77(15.3) | 1.11(0.80,1.56) | 0.536 | 88(22.9) | 117(32.1) | **2.05(1.36,3.08)** | **0.001** |
| Chemotherapy |  |  |  |  |  |  |  |  |
| No | 1897(75.1) | 304(60.4) | 1(ref) |  | 88(22.9) | 108(29.7) | 1(ref) |  |
| Yes | 629(24.9) | 199(39.6) | **1.76(1.31,2.37)** | **<0.001** | 296(77.1) | 256(70.3) | 0.92(0.60,1.40) | 0.694 |
| Radiotherapy |  |  |  |  |  |  |  |  |
| No | 944(37.4) | 164(32.6) | 1(ref) |  | 128(33.3) | 119(32.7) | 1(ref) |  |
| Yes | 1582(62.6) | 339(67.4) | **1.32(1.04,1.68)** | **0.022** | 256(66.7) | 245(67.3) | 1.05(0.72,1.53) | 0.818 |
| Surgery |  |  |  |  |  |  |  |  |
| No | 117(4.6) | 21(4.2) | 1(ref) |  | 27(7.0) | 16(4.4) | 1(ref) |  |
| Yes | 2409(95.4) | 482(95.8) | 1.32(0.58,3.03) | 0.510 | 357(93.0) | 348(95.6) | 0.99(0.35,2.84) | 0.994 |

Note: FPBC, first primary breast cancer; SPBC, second primary breast cancer. *, all index variables were initially included in the multivariate logistic regression analyses. ER, estrogen receptor; HER2, human epidermal growth factor receptor-2.

**Table S6. Multivariate** **logistic analyses on the factors associated with change of PR status.**

| Characteristics of  FPBC* | FPBC (+), N (%) | | OR (95%CI) | *P* value | FPBC (-), N (%) | | OR (95%CI) | *P* value |  |
| --- | --- | --- | --- | --- | --- | --- | --- | --- | --- |
|  | SPBC(+) (Ref.) | SPBC(-) |  |  | SPBC(+) | SPBC(-) (Ref.) |  |  |  |
| Age at diagnosis |  |  |  |  |  |  |  |  | |
| <50 years | 417(23.6) | 177(19.6) | 1(ref) |  | 177(25.8) | 73(17.5) | 1(ref) |  | |
| 50-60 years | 372(21.0) | 233(25.8) | **1.76(1.33,2.34)** | **<0.001** | 215(31.4) | 111(26.6) | 1.27(0.82,1.95) | 0.286 | |
| ≥ 60 years | 981(55.4) | 494(54.7) | **1.55(1.20,2.00)** | **0.001** | 293(42.8) | 234(56.0) | **1.92(1.28,2.86)** | **0.002** | |
| Race |  |  |  |  |  |  |  |  | |
| White | 1401(79.2) | 675(74.8) | 1(ref) |  | 425(62.2) | 326(78.2) | 1(ref) |  | |
| Non-white | 368(20.8) | 228(25.3) | **1.27(1.02,1.59)** | **0.034** | 258(37.8) | 91(21.8) | **0.47(0.34,0.66)** | **<0.001** | |
| Grade |  |  |  |  |  |  |  |  | |
| I+II | 1381(82.4) | 641(74.5) | 1(ref) |  | 58(33.1) | 153(38.8) | 1(ref) |  | |
| III | 294(17.6) | 219(25.5) | 1.25(0.98,1.59) | 0.076 | 427(66.9) | 241(61.2) | 0.86(0.63,1.19) | 0.364 | |
| SEER historic stage |  |  |  |  |  |  |  |  | |
| In situ + localized | 1357(76.9) | 620(68.9) | 1(ref) |  | 425(62.2) | 275(66) | 1(ref0 |  | |
| Regional + distant | 408(23.1) | 280(31.1) | 1.02(0.75,1.40) | 0.884 | 258(37.8) | 142(34) | 0.89(0.60,1.32) | 0.562 | |
| HER2 status |  |  |  |  |  |  |  |  | |
| Negative | 1621(91.6) | 780(86.3) | 1(ref) |  | 518(75.6) | 292(69.9) | 1(ref) |  | |
| Positive | 149(8.4) | 124(13.7) | **1.46(1.06,2.02)** | **0.020** | 167(24.4) | 126(30.1) | **1.83(1.31,2.55)** | **<0.001** | |
| Chemotherapy |  |  |  |  |  |  |  |  | |
| No | 1388(78.4) | 600(66.4) | 1(ref) |  | 228(33.3) | 181(43.3) | 1(ref) |  | |
| Yes | 382(21.6) | 304(33.6) | **1.61(1.23,2.12)** | **0.001** | 457(66.7) | 237(56.7) | **0.69(0.49,0.96)** | **0.026** | |
| Radiotherapy |  |  |  |  |  |  |  |  | |
| No | 672(38.0) | 298(33.0) | 1(ref) |  | 237(34.6) | 148(35.4) | 1(ref) |  | |
| Yes | 1098(62.0) | 606(67.0) | **1.24(1.02,1.52)** | **0.033** | 448(65.4) | 270(64.6) | 1.05(0.77,1.44) | 0.757 | |
| Surgery |  |  |  |  |  |  |  |  | |
| No | 73(4.1) | 46(5.1) | 1(ref) |  | 42(6.1) | 20(4.8) | 1(ref) |  | |
| Yes | 1697(95.9) | 858(94.9) | 1.10(0.57,2.10) | 0.782 | 643(93.9) | 398(95.2) | 0.97(0.37,2.55) | 0.952 | |

Note: FPBC, first primary breast cancer; SPBC, second primary breast cancer. *, all index variables were initially included in the multivariate logistic regression analyses. PR, progesterone receptor; HER2, human epidermal growth factor receptor-2.

**Table S7. Multivariate** **logistic analyses on the factors associated with change of HER2 status.**

| Characteristics of  FPBC* | FPBC (+), N (%) | | OR (95%CI) | P value | FPBC (-), N (%) | | OR (95%CI) | *P* value |
| --- | --- | --- | --- | --- | --- | --- | --- | --- |
|  | SPBC(+) (Ref.) | SPBC(-) |  |  | SPBC(+) | SPBC(-) (Ref.) |  |  |
| Age at diagnosis |  |  |  |  |  |  |  |  |
| <50 years | 96(39.2) | 72(22.4) | 1(ref) |  | 594(20.9) | 82(22.2) | 1(ref) |  |
| 50-60 years | 82(33.5) | 88(27.4) | 1.55(0.92,2.62) | 0.101 | 659(23.2) | 102(27.6) | 1.07(0.76,1.51) | 0.711 |
| ≥ 60 years | 67(27.4) | 161(50.2) | **3.66(2.16,6.19)** | **<0.001** | 1588(55.9) | 186(50.3) | 0.76(0.55,1.05) | 0.092 |
| Race |  |  |  |  |  |  |  |  |
| White | 170(69.7) | 219(68.4) | 1(ref) |  | 2159(76.1) | 279(75.4) | 1(ref) |  |
| Non-white | 74(30.3) | 101(31.6) | 1.04(0.65,1.64) | 0.880 | 679(23.9) | 91(24.6) | 1.02(0.77,1.37) | 0.870 |
| Grade |  |  |  |  |  |  |  |  |
| I+II | 84(39.0) | 124(41.7) | 1(ref) |  | 1915(70.9) | 264(73.9) | 1(ref) |  |
| III | 130(61.0) | 173(58.3) | 0.98(0.62,1.53) | 0.915 | 785(29.1) | 93(26.1) | 0.92(0.67,1.28) | 0.635 |
| SEER historic stage |  |  |  |  |  |  |  |  |
| In situ + localized | 124(51.2) | 207(64.5) | 1(ref) |  | 2053(72.5) | 293(79.2) | 1(ref) |  |
| Regional + distant | 118(48.8) | 114(35.5) | 0.61(0.32,1.18) | 0.144 | 779(27.5) | 77(20.8) | **0.61(0.41,0.91)** | **0.015** |
| HR status |  |  |  |  |  |  |  |  |
| Negative | 81(33.1) | 112(34.9) | 1(ref) |  | 466(16.4) | 48(13.0) | 1(ref) |  |
| Positive | 164(66.9) | 209(65.1) | 0.93(0.58,1.47) | 0.742 | 2375(83.6) | 322(87.0) | 1.39(0.92,2.12) | 0.121 |
| Chemotherapy |  |  |  |  |  |  |  |  |
| No | 97(39.6) | 127(39.6) | 1(ref) |  | 1913(67.3) | 260(70.3) | 1(ref) |  |
| Yes | 148(60.4) | 194(60.4) | 1.13(0.69,1.88) | 0.626 | 928(32.7) | 110(29.7) | 1.10(0.77,1.56) | 0.603 |
| Radiotherapy |  |  |  |  |  |  |  |  |
| No | 93(38.0) | 125(38.9) | 1(ref) |  | 1018(35.8) | 119(32.2) | 1(ref) |  |
| Yes | 152(62.0) | 196(61.1) | 0.81(0.52,1.26) | 0.346 | 1823(64.2) | 251(67.8) | 1.12(0.87,1.46) | 0.381 |
| Surgery |  |  |  |  |  |  |  |  |
| No | 31(12.7) | 20(6.2) | 1(ref) |  | 123(4.3) | 7(1.9) | 1(ref) |  |
| Yes | 214(87.4) | 301(93.8) | 1.78(0.63,5.05) | 0.278 | 2718(95.7) | 363(98.1) | 1.75(0.53,5.72) | 0.358 |

Note: FPBC, first primary breast cancer; SPBC, second primary breast cancer. *, all index variables were initially included in the multivariate analyses. HER2, human epidermal growth factor receptor-2; HR, hormone receptor.

**Table S8. Clinical characteristics between included patients and excluded patients.**

| Clinical characteristics | Overall FPBC  patients | FPBC patients  without SPC | SPC patients  excluding SPBC | SPBC  patients | *P*  *value^1^* | *P*  *value^2^* |
| --- | --- | --- | --- | --- | --- | --- |
|  | (N=362789) | (N=347508) | (N=11504) | (N=3777) |  |  |
| Age, mean (SD) | 59.17 (12.39) | 59.00 (12.39) | 64.04 (11.37) | 60.00 (12.70) | <0.001 | <0.001 |
| Race, N (%) |  |  |  |  | <0.001 | <0.001 |
| White | 281642 (77.6) | 269433 (77.5) | 9382 (81.6) | 2827 (74.8) |  |  |
| Others | 78511 (21.6) | 75457 (21.7) | 2109 (18.3) | 945 (25.0) |  |  |
| Unknown | 2636 (0.7) | 2618 (0.8) | 13 (0.1) | 5 (0.1) |  |  |
| Marital, N (%) |  |  |  |  | <0.001 | 0.134 |
| Married | 206273 (56.9) | 198134 (57.0) | 6078 (52.8) | 2061 (54.6) |  |  |
| Others | 138017 (38.0) | 131626 (37.9) | 4864 (42.3) | 1527 (40.4) |  |  |
| Unknown | 18499 (5.1) | 17748 (5.1) | 562 (4.9) | 189 (5.0) |  |  |
| Year of diagnosis, N (%) |  |  |  |  | <0.001 | <0.001 |
| 2010 | 38148 (10.5) | 34973 (10.1) | 2287 (19.9) | 888 (23.5) |  |  |
| 2011 | 40504 (11.2) | 37565 (10.8) | 2142 (18.6) | 797 (21.1) |  |  |
| 2012 | 41515 (11.4) | 38918 (11.2) | 1925 (16.7) | 672 (17.8) |  |  |
| 2013 | 42793 (11.8) | 40652 (11.7) | 1672 (14.5) | 469 (12.4) |  |  |
| 2014 | 43324 (11.9) | 41679 (12.0) | 1280 (11.1) | 365 (9.7) |  |  |
| 2015 | 44553 (12.3) | 43276 (12.5) | 1007 (8.8) | 270 (7.1) |  |  |
| 2016 | 44777 (12.3) | 43875 (12.6) | 709 (6.2) | 193 (5.1) |  |  |
| 2017 | 44550 (12.3) | 44011 (12.7) | 430 (3.7) | 109 (2.9) |  |  |
| 2018 | 22625 (6.2) | 22559 (6.5) | 52 (0.5) | 14 (0.4) |  |  |
| Laterality, N (%) |  |  |  |  | 0.117 | 0.634 |
| Left | 183737 (50.6) | 175944 (50.6) | 5885 (51.2) | 1908 (50.5) |  |  |
| Right | 178489 (49.2) | 171013 (49.2) | 5609 (48.8) | 1867 (49.4) |  |  |
| Others | 563 (0.2) | 551 (0.2) | 10 (0.1) | 2 (0.1) |  |  |
| Grade, N (%) |  |  |  |  | <0.001 | <0.001 |
| I | 73726 (20.3) | 70151 (20.2) | 2717 (23.6) | 858 (22.7) |  |  |
| II | 143778 (39.6) | 137292 (39.5) | 4958 (43.1) | 1528 (40.5) |  |  |
| III | 107803 (29.7) | 103290 (29.7) | 3332 (29.0) | 1181 (31.3) |  |  |
| IV | 1032 (0.3) | 990 (0.3) | 26 (0.2) | 16 (0.4) |  |  |
| Unknown | 36450 (10.0) | 35785 (10.3) | 471 (4.1) | 194 (5.1) |  |  |
| SEER historical stage, N (%) |  |  |  |  | <0.001 | <0.001 |
| In situ | 8131 (2.2) | 7562 (2.2) | 340 (3.0) | 229 (6.1) |  |  |
| Localized | 229115 (63.2) | 219111 (63.1) | 7556 (65.7) | 2448 (64.8) |  |  |
| Regional | 107673 (29.7) | 103500 (29.8) | 3228 (28.1) | 945 (25.0) |  |  |
| Distance | 15933 (4.4) | 15453 (4.4) | 337 (2.9) | 143 (3.8) |  |  |
| Unknown | 1937 (0.5) | 1882 (0.5) | 43 (0.4) | 12 (0.3) |  |  |
| Radiation (yes), N (%) | 217246 (59.9) | 208109 (59.9) | 6854 (59.6) | 2283 (60.4) | 0.625 | 0.356 |
| Surgery (yes), N (%) | 339517 (93.6) | 324894 (93.5) | 11027 (95.9) | 3596 (95.2) | <0.001 | 0.099 |
| Chemotherapy (yes), N (%) | 156663 (43.2) | 151136 (43.5) | 4147 (36.0) | 1380 (36.5) | <0.001 | 0.601 |
| Summary subtype, N (%) |  |  |  |  | <0.001 | <0.001 |
| HR+/HER2- | 262026 (72.2) | 250607 (72.1) | 8722 (75.8) | 2697 (71.4) |  |  |
| HR+/HER2+ | 42096 (11.6) | 40662 (11.7) | 1061 (9.2) | 373 (9.9) |  |  |
| HR-/HER2- | 40756 (11.2) | 38970 (11.2) | 1272 (11.1) | 514 (13.6) |  |  |
| HR-/HER2+ | 17911 (4.9) | 17269 (5.0) | 449 (3.9) | 193 (5.1) |  |  |
| ER (+), N (%) | 300382 (82.8) | 287665 (82.8) | 9688 (84.2) | 3029 (80.2) | <0.001 | <0.001 |
| PR (+), N (%) | 263381 (72.6) | 252229 (72.6) | 8478 (73.7) | 2674 (70.8) | 0.001 | <0.001 |
| HER2 (+), N (%) | 60007 (16.5) | 57931 (16.7) | 1510 (13.1) | 566 (15.0) | <0.001 | <0.001 |
| Registry, N (%) |  |  |  |  | <0.001 | <0.001 |
| Alaska Natives | 431 (0.1) | 431 (0.1) | 0 (0.0) | 0 (0.0) |  |  |
| California | 145106 (40.0) | 139217 (40.1) | 4374 (38.0) | 1515 (40.1) |  |  |
| Connecticut | 17491 (4.8) | 16684 (4.8) | 623 (5.4) | 184 (4.9) |  |  |
| Detroit (Metropolitan) | 18119 (5.0) | 17294 (5.0) | 612 (5.3) | 213 (5.6) |  |  |
| Georgia | 42095 (11.6) | 40359 (11.6) | 1318 (11.5) | 418 (11.1) |  |  |
| Hawaii | 7060 (1.9) | 6757 (1.9) | 199 (1.7) | 104 (2.8) |  |  |
| Iowa | 14448 (4.0) | 13741 (4.0) | 539 (4.7) | 168 (4.4) |  |  |
| Kentucky | 19795 (5.5) | 18912 (5.4) | 686 (6.0) | 197 (5.2) |  |  |
| Louisiana | 20076 (5.5) | 19170 (5.5) | 655 (5.7) | 251 (6.6) |  |  |
| New Jersey | 39983 (11.0) | 38314 (11.0) | 1319 (11.5) | 350 (9.3) |  |  |
| New Mexico | 7501 (2.1) | 7221 (2.1) | 219 (1.9) | 61 (1.6) |  |  |
| Seattle (Puget Sound) | 21858 (6.0) | 20944 (6.0) | 681 (5.9) | 233 (6.2) |  |  |
| Utah | 8826 (2.4) | 8464 (2.4) | 279 (2.4) | 83 (2.2) |  |  |

Note: FPBC, first primary breast cancer; SPC, second primary cancer; SPBC, second primary breast cancer; ER, estrogen receptor; PR, progesterone receptor; HER2, human epidermal growth factor receptor-2. 1, *P* value for chi-square test between all three groups of patients; 2, *P* value for chi-square test between SPC patients excluding SPBC and SPBC patients.
